# Quantitative assays for the measurement of HER1-HER2 heterodimerization and phosphorylation in cell lines and breast tumors: applications for diagnostics and targeted drug mechanism of action

**DOI:** 10.1186/bcr2866

**Published:** 2011-04-15

**Authors:** Lisa DeFazio-Eli, Kristi Strommen, Trang Dao-Pick, Gordon Parry, Laurie Goodman, John Winslow

**Affiliations:** 1 Department of Oncology Research and Development, Monogram Biosciences, Inc., 345 Oyster Point Blvd., South San Francisco, CA 94080, USA

## Abstract

**Introduction:**

Ligand-bound and phosphorylated ErbB/HER heterodimers are potent signaling forms of this receptor family, and quantitative measurements of these active receptors may be predictive of patient response to targeted therapies. Using VeraTag™ technology, we developed and characterized quantitative assays measuring epidermal growth factor (EGF)-dependent increases in activated HER receptors in tumor cell line lysates and formalin-fixed, paraffin-embedded (FFPE) tumor sections. We demonstrated the ability of the assays to quantitatively measure changes in activated HER1 and HER2 receptor levels in cell lines following treatment with 2C4, erlotinib, and lapatinib. We utilized these assays to determine the prevalence and distribution of activated HER1, HER2, and HER1-HER2 heterodimers in 43 HER2-positive breast tumors.

**Methods:**

Assays for activated HER1 and HER2 receptors in FFPE and cell lysate formats were developed using VeraTag™ technology, which requires the proximity of an antibody pair for light-dependent release of a fluorescently labeled tag, followed by capillary electrophoresis-based quantitation.

**Results:**

Ligand-dependent and independent HER1-HER2 heterodimer levels measured by lysate and FFPE VeraTag™ assays trended with HER1 and HER2 expression levels in tumor cell lines, which was confirmed by co-immunoprecipitation. The formation of EGF-dependent HER1-HER2 heterodimers were inhibited by the HER2-targeted monoclonal antibody 2C4 and stabilized by the HER1 tyrosine kinase inhibitor (TKI) erlotinib. EGF-dependent HER1 and HER2 phosphorylation was inhibited by lapatinib and erlotinib. Further, we observed that dominant receptor signaling patterns may switch between HER1-HER1 and HER1-HER2, depending on drug mechanism of action and relative levels of HER receptors. In FFPE breast tumors that expressed both HER1 and HER2, HER1-HER2 heterodimers were detected in 25 to 50% of tumors, depending on detection method. The levels of activated phospho-HER1-HER2 heterodimers correlated with HER1 or HER2 levels in an analysis of 43 HER2-positive breast tumors.

**Conclusions:**

VeraTag™ lysate assays can be used as a tool for understanding the mechanism of action of targeted HER-family inhibitors in the preclinical setting, while VeraTag™ FFPE assays of activated HER receptors combined with total HER2 measurements (HERmark^®^) in tumor samples may provide a more accurate prediction of clinical response to both HER1 and HER2 targeted therapies.

## Introduction

Both the epidermal growth factor receptor (EGFR/HER1) and HER2 are members of the ErbB family of the type I receptor tyrosine kinases, which also includes HER3 and HER4. These homologous receptors are comprised of an extracellular binding domain (ECD), a transmembrane domain, and an intracellular tyrosine kinase (TK) domain. Binding of ligand to the ECD induces structural reorganization allowing for functional homo- and heterodimerization and activation of the kinase domain [1-3]. HER1 has several ligands including EGF, transforming growth factor α, amphiregulin, betacellulin, epiregulin and heparin binding-EGF [4-7]. A HER2 ligand has not been identified, but overexpressed HER2 is constitutively active [8]. In cells expressing both HER1 and HER2, binding of ligand to HER1 can induce HER1-HER1 homodimerization and HER1-HER2 heterodimerization. These active dimers transmit through signaling pathways including Ras/Raf/MEK/ERK and PI3K/Akt, which are important for tumor growth and metastasis [9]. Recent studies have shown that HER1-HER1 homodimers and HER1-HER2 heterodimers also exist in inactive, non-ligand bound conformations which may structurally rearrange upon ligand binding to form actively signaling complexes [10-14].

HER2 overexpression has been observed in several cancer types [15]. From 15 to 30% of human breast tumors display HER2 gene amplification or protein overexpression, which is prognostic for poor outcome and predictive of a response to trastuzumab [16,17]. HER1 overexpression has also been observed in colorectal, gastric, breast, ovarian, non-small cell lung, and head and neck carcinomas as well as glioblastoma [15] and has been shown to contribute to cellular transformation and proliferation [18,19]. Potential cooperativity of HER1 and HER2 in mouse mammary tumorigenesis has been reported [20,21]. Furthermore, human breast and ovarian tumors that overexpress both HER1 and HER2 may have a less favorable outcome [22,23]. Finally, a retrospective immunohistochemical analysis of 807 FFPE breast tumor samples showed that patients whose tumors expressed phosphorylated HER2 or co-expressed HER1 and HER2 had the shortest survival [24]. These studies support a potential role for HER1 signaling in breast cancer.

Several drugs that target HER1 and HER2 receptors have been utilized in both preclinical and clinical models of breast and other cancers. Treatment with the humanized monocolonal HER2 antibody trastuzumab is now standard of care for individuals with HER2-positive invasive breast cancer in both the metastatic and adjuvant settings. However, fewer than 50% of patients with metastatic HER2-positive breast tumors show initial benefit from trastuzumab treatment, and many of those eventually develop resistance [25-27]. Thus, exclusive measurement of total HER2 receptor level may not provide sufficient information for prediction of drug response. Recent studies have indicated that stratifying HER2 positive breast cancer patients by HER3 or p95 protein expression can result in the identification of multiple sub-populations of patients that exhibit significant differences in time to progression following trastuzumab treatment [28,29].

Preclinical studies in human breast cancer cell lines selected for trastuzumab resistance both *in vitro *and in xenograft models have demonstrated overexpression of HER1 and its ligands [30]. Further *in vitro *studies have shown that stable transfection and expression of HER1 in the background of a HER2-positive cell line induces trastuzumab resistance [31]. HER1 up-regulation has also been observed in estrogen-receptor positive breast cancers that develop resistance to treatment with tamoxifen [32,33]. *In vitro *selection of the human breast cancer cell line MCF7 for tamoxifen resistance resulted in up-regulation of HER1, and increased HER1-HER2 heterodimerization and phosphorylation [34]. Drugs that specifically target active HER dimerization or phosphorylation have been investigated both preclinically and in clinical trials. Three such drugs are pertuzumab (humanized 2C4), which inhibits HER2-containing heterodimers; erlotinib, a HER1 tyrosine kinase inhibitor (TKI), and lapatinib, a dual HER1/HER2 TKI.

Resistance to trastuzumab and the emergence of multiple new HER1 and HER2-targeted therapies support a need for assays which directly measure not only total amounts of HER1 and HER2 receptors, but more specifically HER1 and HER2 activation. The ability to selectively detect and quantify HER1 and HER2 dimerization and phosphorylation may facilitate a better understanding of drug mechanism of action. Such assays may have the potential to predict drug response. Traditional methods to measure dimerization rely upon low throughput, qualitative technologies such as co-immunoprecipitation and cross-linking, which require cellular lysates, whereas the standard tumor format used for patient testing is FFPE tissue. Phosphorylated receptors are also measured in lysates by co-immunoprecipitation/Western blotting, or semi-quantitatively in FFPE sections using immunohistochemistry (IHC). Recently, a proximity ligation assay (PLA) has been utilized to detect epitope-tagged and ligand-dependent HER1 homodimers in fixed cells overexpressing transfected HER1 [35], and native HER1-HER2 heterodimers by fluorescent activated cell sorting (FACS) [36]. However, there is limited characterization of the PLA assay in FFPE tumor tissue [37]. VeraTag™ technology, which utilizes a fluorescence-based dual antibody system, can be utilized to specifically and quantitatively measure protein-protein interactions [38], and is currently used in the commercially available HERmark^® ^assay, which measures total HER2 and HER2-HER2 associations in FFPE breast tumors [39]. Here, we utilized VeraTag™ technology to develop assays that measure HER1 and HER2 dimerization and phosphorylation in both lysate and FFPE formats. We demonstrated the potential utility of such assays to characterize the effects of 2C4, erlotinib, and lapatinib on HER1 and HER2 activation and determined the prevalence of HER1-HER2 heterodimers and activated forms in 43 HER2-positive breast tumors. These assays have the potential to characterize targeted drugs in pre-clinical studies and identify predictive biomarkers that could guide personalized treatment decisions.

## Materials and methods

### Cell line growth and EGF stimulation

Cell lines AU565, SKBR3, SKOV3, H1650, MDA-MB-453, and MDA-MB-468 were purchased from American Type Culture Collection (ATCC, Manassas, VA, USA) and cultured in either McCoy's 5A Modified (SKOV3 and SKBR3; Lonza, Basel, Switzerland), RPMI-1640 (AU565, H1650; Lonza), or DMEM (MDA-MB-453, MDA-MB-468, Gibco, Carslbad, CA, USA) media, supplemented with 10% heat-inactivated fetal bovine serum (1X L-Glutamine, and 1X penicillin-streptomycin (Gibco). EGF was purchased from Peprotech (Rocky Hill, NJ, USA).

For EGF stimulation experiments, cells were grown in cell line-specific media to approximately 80% confluence. Cells were washed thrice with phosphate-buffered saline (PBS) and starved in serum-free media for 18 hours. The next morning, cells were briefly washed in PBS and exposed to fresh serum-free media for another 25 minutes, followed by a final exposure to fresh serum-free media containing EGF at the indicated concentration for 10 minutes at 37°C. To terminate EGF stimulation, cells were placed immediately on ice and were washed briefly two times with ice-cold PBS. Subsequently, the cells were processed for either lysate or FFPE.

### Transfected 293 clones

A total of 293 cells were transfected with pcDNA3.1-Zeocin-HER1 plasmids per Fugene6 Transfection Reagent protocol (Roche Indianapolis, IN, USA). Individual clones were isolated and Western blot and flow cytometry analyses were performed to confirm the presence of HER1 proteins. 293H1clone11 was further transfected with pcDNA-Neocin containing HER2 cDNA sequence using the method described above. 293H1H2 clones 15, 16, and 19 displayed similar levels of HER1 as 293H1c11 and different levels of HER2 by flow cytometry.

### Preparation of lysate and FFPE samples

For lysate preparation, the cells were scraped in ice cold PBS containing phosphatase and protease inhibitors (50 mM NaF, 50 mM beta-glycerophosphate, 1 mM Na_3_V0_4_, and one protease inhibitor tablet (Roche, Indianapolis, IN, USA) per 10 mL, and centrifuged (five minutes, 1,000 rpm, 4°C). Supernatant was decanted and the cell pellet was lysed on ice for 30 minutes in lysis buffer (1% Triton X-100, 50 mM Tris-HCl, pH 8.0, 150 mM NaCl) containing phosphatase and protease inhibitors. Lysates were centrifuged (10 minutes, 10,000 rpm, 4°C) to separate insoluble material. Total protein concentration was measured using bicinchonicic acid reagent (Pierce, Rockford, IL, USA). Lysates were stored at -80°C.

For FFPE preparation, the cells were fixed on the tissue culture plate in 10% Neutral Buffered Formalin (Richard-Allan Scientific, Kalamazoo, MI, USA) for 30 minutes at 4°C, after which time they were scraped and centrifuged (5 minutes, 3,000 rpm, 4°C). Supernatant was decanted and cell pellets were packed for paraffin-embedding (Tissue-Tek). FFPE samples were sectioned at 5 μm onto positively charged microscope slides (VWR, West Chester, PA, USA) and stored at 4°C. Macrodissection of FFPE sections to be analyzed by the HER1 VeraTag™ assay was performed to obtain sections with >70% tumor.

All breast tumor tissues were acquired from a single commercial vendor (Asterand, Detroit, MI, USA), which was chosen as our supplier because of their rigorous controls around tissue collection, preparation and storage. Specifically, all tissues (median tumor length: 7 mm) were fixed or snap-frozen within 15 to 30 minutes of excision. All tissues were fixed identically in neutral-buffered formalin as dictated by the vendor's Standard Operating Procedure, which is consistent with the ASCO/CAP guidelines for preparation of breast tumor tissue for HER2 testing.

### Drug treatment

2C4 (Hybridoma from ATCC) was dissolved in sterile PBS at a stock concentration of 1.73 mg/mL. Lapatinib (American Custom Chemical Corporation, San Diego, CA, USA) and erlotinib (extracted from tablets as described in [40]) were each dissolved in dimethyl sulfoxide (DMSO) at a stock concentration of 5 mM and 10 mM, respectively. All compounds were stored at -80°C until use. Cells were serum starved overnight and re-serum starved the next morning as described above prior to exposure to either 2C4 (2 μg/mL or 20 μg/mL), lapatinib (0.1 μM or 1 μM), erlotinib (0.1 μM or 1 μM), or carrier solution (1 × PBS or DMSO) for two hours at 37°C. Cells were then exposed or mock-exposed to 16 nM EGF for 10 minutes at 37°C. EGF stimulation was terminated by washing with ice cold PBS and cells were processed either for lysate or FFPE preparation as described above.

### Antibodies and peptides

All antibodies used in VeraTag™ assays and co-immunoprecipitation (co-IP)/Western blotting experiments were monoclonal. HER2-Ab8 and HER2-Ab-15 (LabVision, Fremont, CA, USA) were directed against the intracellular domain of HER2. HER2-Ab4 and HER2-Ab5 (LabVision) were directed against the ECD of HER2. HER2-Ab18 (Clone PN2A) was directed against phosphotyrosine 1248 of HER2 (LabVision). Clone 6B12 was directed against phosphotyrosines 1221 and 1222 of HER2 (Cell Signaling, Danvers, MA, USA). HER1-Ab15 (LabVision) was directed against the intracellular domain of HER1. HER1-Ab11 and HER1-Ab5 (LabVision) were directed against the HER1 ECD. Clones 53A5 (Cell Signaling) and 1H12 (Cell Signaling) were directed against HER1 phosphotyrosines 1173 and 1068, respectively. Clone 4G10 (Millipore, Billerica, MA, USA) was a pan-phosphotyrosine antibody. For VeraTag™ assays, antibodies were conjugated to either biotin or the VeraTag™ fluorescent reporter pro11 (US Patent 7,105,308) as described previously [38]. HER1-HER1 homodimerization was measured using an antibody to a single epitope conjugated to either biotin or a VeraTag™ reporter [38].

Antigenic peptides for HER1-53A5 and HER1-1H12, along with homologous HER2 peptides and non-phosphorylated HER1 and HER2 peptides, were synthesized and HPLC-purified to >95% purity (Biomatik, Cambridge, ON, Canada). Lyophilized peptides were resuspended in nuclease-free water to a concentration of 1 mg/ml.

### VeraTag™ lysate and FFPE assay technology

**Figure 1:**
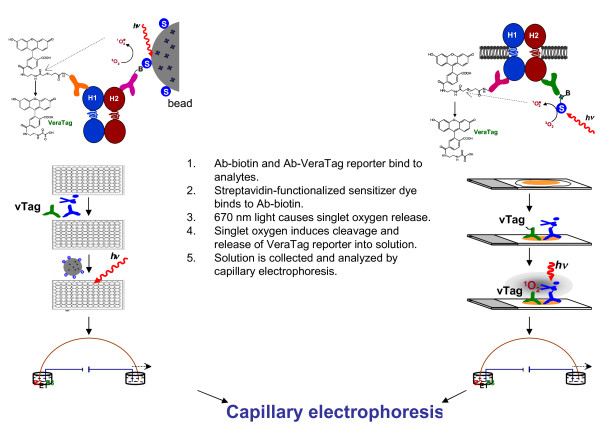
**Depiction of VeraTag™ technology**. In both lysate and FFPE assays, one antibody conjugated to biotin and one antibody conjugated to a VeraTag™ reporter bind to their respective analytes. Streptavidin-functionalized sensitizer dye binds to Ab-biotin. 670 nm light causes singlet oxygen release, which induces cleavage and release of the VeraTag™ reporter molecule into illumination buffer (IB). IB is collected and analyzed by capillary electrophoresis. For details, see Materials and methods.

VeraTag™ assays were performed as described previously [38,39] and as depicted in Figure 1. In brief, VeraTag™ technology relies on the proximity of two antibodies, one conjugated to biotin and the other conjugated to a fluorescent reporter dye, denoted as proN. For lysate assays, both conjugated antibodies were incubated with cell or tumor lysates for one hour prior to addition of streptavidin-coated beads that were infused with the photosensitizer dye, methylene blue. Four to eight serial dilutions of each lysate were run over pre-blocked 96-well filter plates and washed three times to retain the antibody-protein complexes bound to the beads. The retained bead-antibody-protein complexes were then suspended in illumination buffer (IB) containing a fluorescein standard and two additional capillary electrophoresis (CE) mobility markers in 0.01X PBS [39]. Red light (670 nm wavelength) was shined on the solutions using a customized LED illuminator to activate the methylene blue photosensitizer molecule, causing singlet oxygen to cleave the VeraTag™ reporter at its thio-ester bond and release the fluorescent VeraTag™ molecule. Samples were collected and analyzed by CE (Applied Biosystems 3100) using customized eTag Informer software. Measurement of VeraTag™ signals were in units of relative peak area (RPA), which corresponds to the peak height integrated over the elution time of the released VeraTag™ reporter, normalized to that of the internal fluorescein control. Results for each of the serial dilutions were analyzed by linear regression, and were reported as RPA/mg protein.

For FFPE assays, heat-induced epitope retrieval was performed in either Diva Decloaker or Borg Decloaker (Biocare Medical, Concord, CA, USA) in a Decloaking Chamber (BioCare Medical). Slides were washed with water and blocked with blocking buffer (1.5% BSA in 1 × PBS, 1% mouse serum, plus protease and phosphatase inhibitors), then incubated with the conjugated antibodies in blocking buffer overnight. For peptide competition assays, the antigenic peptides were co-incubated overnight with the conjugated antibodies. The next morning, slides were washed once with PBS + 0.25% Triton, then once with PBS. Steptavidin bound to methylene blue (SA-MB, US Patent 7,105,308) in 1 × PBS was added for one hour, and slides were washed several times with PBS + 0.25% Triton and water in the dark prior to addition of IB and exposure to red light for two hours at 4°C using a customized LED illuminator. Slides were then further incubated for one hour at room temperature. IB was collected and VeraTag™ reporters were quantitated by capillary electrophoresis (CE-Applied Biosystems 3100) in duplicate, and RPA was determined with eTag Informer software. For each sample, isotype control (ITC) assays were also performed on an adjacent section, wherein the specific biotin-conjugated antibody was substituted with a biotin-conjugated ITC IgG. Following VeraTag™ FFPE assay, all slides were subjected to hematoxylin and eosin staining and submitted to analysis for tumor content by a board-certified pathologist. Tumor area and section size were quantitated using Image-Pro Plus 6.0 software (Media Cybernetics, Bethesda, MD, USA). Cell line sections were considered to be 100% tumor, and, therefore, the tumor area was equal to section size. Results were reported as normalized RPA, which equals ((RPA*IB volume)/tumor area) of the actual assayed section. In some cases these results were reported with the ITC subtracted. For HER2 total assays, results were reported as HERmark^® ^score [39]. For the HER1-HER2 heterodimer and activated assays, all samples were assayed with a paired isotype control, and positives were classified as those with greater than a low control cell line and with assay signal greater than two-fold over isotype control signal. The two-fold criterion was imposed because all FFPE assay measurements were reproducible within a two-fold window (approximately 20% coefficient of variation); [39]), both within a single batch and between multiple independent batches (data not shown).

Optimal antibody concentrations were identified for each lysate and FFPE assay as follows: Each antibody in each assay was titrated both independently and dependently to assure saturation of epitope in high-expressing samples. Additionally, accuracy was assessed in both ligand-dependent and ligand-independent models and spanned the dynamic range of the HER analytes in the tumor specimens. Detailed explanation of antibody optimization for VeraTag™ assays can be found in [38] and [39].

### Co-immunoprecipitation (Co-IP)/Western blotting

Lysates (250 to 1,000 μg) were pre-cleared by incubation with 40 to 80 μl protein G-sepharose beads (GE Healthcare, Buckinghamshire, UK) for one hour. Antibody was added (either HER2-Ab8 or HER1-Ab15, 2-5 μg) to pre-cleared lysates overnight at 4°C. Protein G-sepharose beads (GE Healthcare) were added and incubated 1.5 hours on a rotator at 4°C. Beads were retained after centrifugation (two minutes, 1,000 rpm, 4°C) and washed twice with 1 × PBS and once with 50 mM Tris-HCL, pH 8.0. Beads were resuspended in Laemmli Sample Buffer (Bio-Rad, Hercules, CA, USA) and incubated at 90°C for 10 minutes. Proteins were separated by electrophoresis using 5% or 7.5% Tris-HCl Ready Gels (Bio-Rad) and transferred to Immuno-Blot PVDF membranes (Bio-Rad). Membranes were blocked for two hours with 5% milk in 1 × PBS + 0.25% Tween 20 (PBS-T) at room temperature. Membranes were incubated overnight at 4°C with the detection antibody in 5% milk + PBS-T. Membranes were washed three times with PBS-T. Goat-anti-mouse-HRP (Bio-Rad, Hercules, CA) or anti-rabbit-HRP (Cell Signaling, Danvers, MA, USA) was then added for two hours, and bands were detected using Supersignal West Pico or Femto substrate (ThermoScientific, Fremont, CA, USA).

### HERI IHC

HER1 IHC was performed by a variation of the Zymed/Invitrogen method, using pepsin (Digest-All, #00-3009; Invitrogen, Carlsbad, CA, USA) and 31G7 monoclonal antibody (#08-4205; Invitrogen). 31G7 concentration was optimized on FFPE cell line controls and tumor sections and used at 0.4 ug/mL for one hour, following pepsin retrieval for 30 minutes at room temperature. All additional steps were performed per manufacturer's protocol.

### Flow cytometry

Cells were harvested by trypsinization and counted. Cells were plated in a 96-well plate and labeled with HER2-Ab4-biotin (Lab Vision) or HER1-Ab11 (Lab Vision) or isotype control (mouse IgG1, BD Biosciences) at a concentration of 4 μg/ml in 100 μl total volume. The cells were incubated with antibody on ice for 45 minutes and washed twice with 1X PBS, followed by labeling with R-Phycoerythrin-Avidin (Invitrogen, Carlsbad, CA) at 2 μg/ml for 30 minutes on ice. The labeled cells were washed twice with 1X PBS and fixed with 1% paraformaldehyde in 1X PBS. Cells were analyzed on a FACSCalibur cytometer (BD Biosciences, San Jose, CA, USA). PE fluorescence intensity of labeled cells was determined on a FL2 (585/42 nm band pass filter) detector. Determination of HER1 or HER2 receptor numbers were based on a calibrated standard curve using Quantum PE MESF Kit (Bangs Laboratories, Inc., Fishers, IN, USA).

## Results

### Identification and characterization of HER1, HER2, and HER1-HER2 heterodimer-expressing cell lines by VeraTag™ lysate assay

In order to measure a HER1 and HER2 heterodimeric complex and associated receptor phosphorylation levels, assays of cell lysates were developed utilizing the VeraTag™ technology (Methods; Figure 1). Preliminarily, several cell lines were identified that contained varying levels of these receptors as measured by flow cytometry (Table 1). To optimize the HER1-HER2 VeraTag™ lysate dimer assay, a matrix of antibodies and conditions were tested for maximal EGF-dependent signal induction in three cell lines that have high levels of HER1 and HER2 expression (SKOV3, SKBR3, and AU565) as measured by flow cytometry. Minimal signal was expected in negative control cell lines MDA-MB-453 and MDA-MB-468, expressing about 5,000 HER1 or 1,000 HER2 receptors per cell, respectively. Time course experiments of ligand activation revealed that 10 to 30 minutes of exposure to either 10 or 100 nM EGF yielded the highest levels of both HER1-HER1 homo- and HER1-HER2 heterodimers (data not shown); therefore, the experiments described here utilized stimulation conditions of 100 nM EGF for 10 minutes (37°C).

**Table 1:** HER1 and HER2 receptor number in the cell line panel

Cell line	HER1	HER2
AU565	204,560	1,447,688
SKBR3	143,599	1,402,832
SKOV3	387,771	657,0880
H1650	158,872	53,810
MDA-MB-453	5,316	292,984
MDA-MB-468	3,389,807	1,209

HER1 and HER2 receptor numbers were determined by FACS analysis. Cells were re-fed with fresh serum-containing media the night prior to harvesting by trypsinization and FACS analysis. HER1 and HER2 receptor number determination was based on a calibrated standard curve.

**Figure 2:**
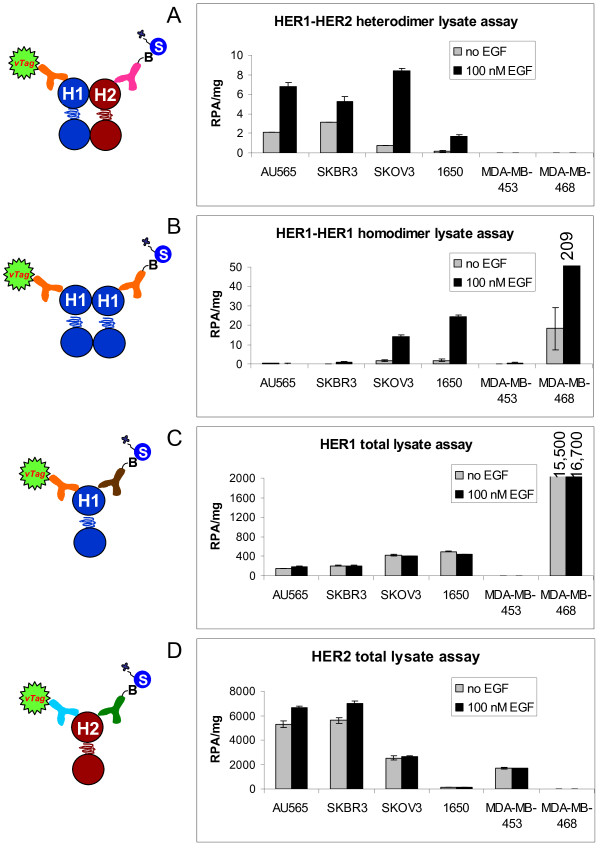
**Cell line panel in VeraTag™ lysate assays**. Cartoons to the left of each graph depict the configuration of the antibodies in the corresponding assay. Experiments were performed using a duplicate eight-point titration of protein input. Points in the linear range were used to calculate RPA/mg protein. Gray bars represent signal from non-stimulated cells and black bars represent stimulation with 100 nM EGF for 10 minutes. A) HER1-HER2 heterodimer. B) HER1-HER1 homodimer. C) HER1 total. D) HER2 total.

Consistent with the overexpresson of HER1 and HER2 as measured by flow cytometry (Table 1), AU565, SKBR3, and SKOV3 cells all displayed high levels of HER1-HER2 heterodimer upon stimulation with EGF (Figure 2a). The cell line H1650, expressing HER1 levels similar to that of SKOV3, but approximately 10-fold lower HER2 expression, showed intermediate HER1-HER2 heterodimer levels upon induction with EGF. No HER1-HER2 signal was apparent in MDA-MB-453 or MDA-MB-468, consistent with these cell lines having very low levels of HER1 or HER2, respectively. All of the cell lines with EGF-induced HER1-HER2 signal also displayed ligand-independent HER1-HER2 signal to varying degrees, consistent with recent reports that HER family receptors may readily form ligand-bound, stable dimers in an active complex, and ligand-independent, less stable dimers as active or inactive complexes [10-14,41,42].

Exposure to EGF can induce formation of both HER1-HER2 heterodimers (Figure 2a) and HER1-HER1 homodimers (Figure 2b) in the six cell lines tested, the relative levels of which depended on the HER1 and HER2 total receptor expression, which were also measured by lysate assays and were unaffected by EGF stimulation (Figures 2c, d). In fact, both ligand-dependent and ligand-independent HER1-HER2 heterodimer signal trended with HER2 levels (Figure 2a), which was also observed in a series of stably transfected HEK293 cells engineered to overexpress similar amounts of HER1 with increasing amounts of HER2 in the same cell background (Figure S1 in Additional File 1). Taken together, these data demonstrate the ability of VeraTag™ assays to quantitatively measure levels of HER1 hetero- and homodimers in tumor cell line lysates, and indicate a dependency of HER1-HER2 heterodimers on the levels of HER1 and HER2.

### Development of HER1-HER2 VeraTag™ FFPE assays

**Figure 3:**
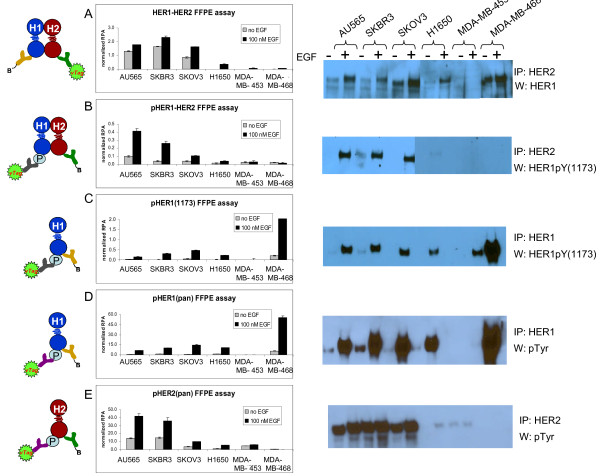
**Cell line panel in HER1-HER2 suite of activated FFPE assays and co-IP**. Cell lines were mock-stimulated or stimulated with 10 nM EGF for 10 minutes, then harvested and either lysed or fixed and paraffin-embedded. Cartoons to the left of each graph depict the configuration of the antibodies in the corresponding VeraTag™ FFPE assay. Gray bars represent normalized RPA from unstimulated and black bars represent signal from EGF-stimulated cells. Duplicate CE injections were measured. Corresponding co-IP assays are to the right of each FFPE assay and all cell lines were assayed in the same experiment. **A) **HER1-HER2. **B) **pHER1-HER2. **C) **pHER1(1173). **D) **pHER1(pan). <**E) **pHER2(pan).

In order to evaluate HER1-HER2 activated forms as potential biomarkers of targeted drug response, cell lines characterized by flow cytometry and lysate assays just described were utilized to develop VeraTag™ assays that measure HER1-HER2 heterodimerization and activation in FFPE tumor tissue (Figure 3a). HER1 and HER2 antibodies were identified and conditions optimized for the highest signal to background detection of HER1-HER2 heterodimers. The HER1 and HER2 antibodies were previously shown to be specific by Western blot and by individual HER1 [38] and HER2 (HERmark^®^) FFPE assays [39]. HER1-HER2 heterodimer assays of FFPE AU565, SKBR3, and SKOV3 cells all displayed specific signal two- to four-fold above an isotype control background (Data not shown, Materials and methods). Negative control cell lines MDA-MB-453 and MDA-MB-468 lacked specific HER1-HER2 signal (Figure 3). Consistent with the HER1-HER2 VeraTag™ lysate assay, 1.5- to -3-fold EGF-dependent increases over ligand-independent signal was observed (Figures 2 and 3). This assay format was confirmed by co-IP/Western blots, in which AU565, SKBR3, SKOV3, and H1650 showed an increase in HER1-HER2 dimerization while MDA-MB-453 was negative. Control experiments indicate that the strong bands detected for stimulated and unstimulated MDA-MB-468 were a result of nonspecific binding of the excessive amounts of expressed HER1 to Protein G beads (data not shown).

In order to discern active HER1-HER2 complexes, FFPE assays that detected phosphorylated forms of HER1, HER2, and the HER1-HER2 heterodimeric complex were developed. Based on target specificity, assay dynamic range, and effects of EGF stimulation, the optimal antibody pairing for measurement of activated HER1-HER2 heterodimers utilized a HER2-specific antibody together with a HER1 antibody specific for the kinase domain phosphotyrosine 1173 to produce a pHER1-HER2 assay (Figure 3b). An EGF-dependent stimulation range of two- to six-fold was observed for activated pHER1-HER2 heterodimers in the FFPE positive controls cell lines, whereas the negative control cells did not display EGF-dependent differences. Similar relative levels of ligand-activated complexes or negative controls were confirmed by co-IP/Western blots (Figure 3b). Since HER1 and HER2 kinase domains share extensive homology, including the HER1 pY1173 and corresponding HER2 pY1248 regions, we confirmed that the monoclonal 53A5 antibody utilized in the pHER1-HER2 assay was specific to phosphorylated HER1 using peptide competition assays (Figure S2 in Additional File 1). Taken together, these results support the antibody specificity of the pHER1-HER2 VeraTag™ assay in FFPE cells.

HER1 and HER2 may be phosphorylated due to actively signaling HER1-HER2 heterodimers, but other mechanisms such as HER1-HER1 dimerization, HER2 autophosphorylation, or via dimerization with other HER family members may also play a role. Thus, assays were designed that could independently assess phosphorylated HER1 and phosphorylated HER2 in FFPE tumor cells. Two phospho-HER1 (pHER1) assays were developed, both of which utilized a HER1 antibody that was matched with either an antibody to measure a specific HER1 phosphotyrosine site (pY1173; clone 53A5), or a pan-phosphotyrosine antibody to measure general HER1 and HER1-associated phosphorylation (pY: clone 4G10). Both the pHER1(1173) and the pHER1(pan) assay formats led to similar trending of pHER1 signal from the cell line panel, consistent with results from co-IP/Western blots (Figure 3c, d). The similar assay profiles of the specific and pan-HER1 phosphorylation illustrate the specificity imparted by the dual antibody system; 4G10 specifically detected HER1 phosphotyrosine due to proximity to the specific HER1 antibody.

To measure the levels of activated HER2, a phosphorylated HER2 assay was developed by pairing a HER2 antibody with the pan-phosphotyrosine antibody 4G10 (Figure 3e). Intriguingly, the EGF-induced signal from the pHER2 assays trended with that of the pHER1-HER2 assay. Since the 53A5 antibody did not cross-react with phospho-HER2, and since HER1 receptor levels in AU565, SKBR3, SKOV3, and H1650 were within two-fold of each other (Table 1), the differences in HER1-HER2 activation were driven primarily by the amount of HER2 in these cell lines.

### Application of VeraTag™ technology to elucidate drug mechanism of action

**Figure 4:**
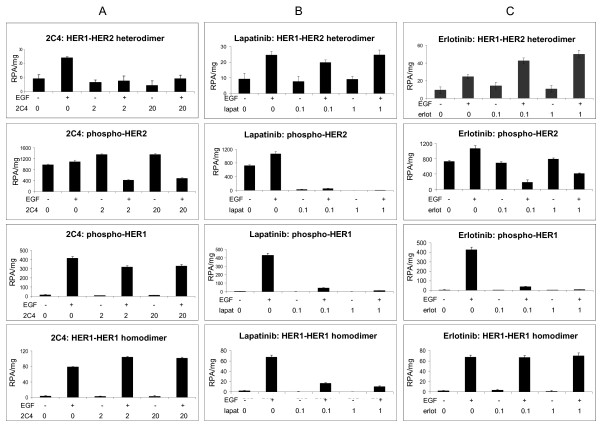
**Effects of HER1-HER2 axis inhibitors measured by VeraTag™ lysate assays**. SKOV3 cells were serum-starved overnight then treated with 2C4, erlotinib, or lapatinib for two hours at the indicated concentrations, in units of μg/mL for 2C4 and in μM for erlotinib and lapatinib. A final concentration of 16 nM EGF was added or mock-added to the drug-containing media for 10 minutes prior to harvesting cells for lysate or FFPE. VeraTag™ lysate assays were used to examine **A) **effects of 2C4, **B) **effects of lapatinib and **C) **effects of erlotinib on HER1-HER2 heterodimerization (top row), phosphorylation of HER2 (second row), phosphorylation of HER1 (third row), and HER1-HER1 homodimerization (bottom row). Each experiment, beginning with a new growth of SKOV3 cells, was performed twice with consistent results.

The direct measurement of ligand-independent and -dependent HER dimers and phosphorylated forms by the VeraTag™ assays allowed the mechanistic characterization of the acute effects (time = 2 hours) of the HER-targeted drugs 2C4, lapatinib, and erlotinib. Cell stimulations were performed with 16 nM EGF instead of 100 nM EGF, which may more closely approximate physiologic conditions while ensuring the same level of dimerization response. Exposure of 2 or 20 ug/mL 2C4, a HER2 monoclonal antibody which inhibits HER2-HER3 heterodimerization [43], decreased EGF-dependent HER1-HER2 heterodimer formation with a small concomitant increase in HER1-HER1 homodimerization (Figure 4a). 2C4 had no significant effect on ligand-independent HER1-HER2 heterodimerization. EGF-dependent HER2 phosphorylation was diminished by three-fold, while HER2 phosphorylation in the absence of EGF was unaffected. A small decrease in EGF-dependent HER1 phosphorylation was observed. In SKBR3, with higher HER2 levels, 20 μg/ml of 2C4 was required to observe loss of HER1-HER2 heterodimerization (data not shown). These results are consistent with 2C4 being an inhibitor of ligand-activated HER2 heterodimerization and phosphorylation but not of ligand-independent HER2 heterodimerization. Furthermore, VeraTag™ measurements also revealed a modest increase in ligand-and 2C4-dependent HER1 homodimers that may result from an increased pool of free HER1 that is no longer able to associate with HER2. A HER2-HER3 VeraTag™ lysate assay [31] confirmed that the concentrations of 2C4 used here caused loss of heregulin-induced HER2-HER3 heterodimerization in MCF7 cells, a well-characterized activity of 2C4 (data not shown) [42,44,45].

As expected, lapatinib, a dual HER1 and HER2 tyrosine kinase inhibitor, nearly eliminated both HER1 and HER2 phosphorylation in SKOV3 cells (Figure 4b). Interestingly, acute treatment with lapatinib also caused loss of EGF-dependent HER1-HER1 homodimerization, but did not affect HER1-HER2 heterodimization in this cell line. Since lapatinib has high affinity to HER2, the effects of lapatinib were tested on H1650 cells that have a similar number of HER1 receptors but approximately 10-fold less HER2 than SKOV3. In H1650 cells a decrease in HER1-HER2 heterodimerizaton but not HER1-HER1 homodimerization was detected (Figure S3A in Additional file 1). The decreased inhibition of HER1 homodimerization in H1650 cells is consistent with an observed residual HER1 phosphorylation. The insensitivity of HER1-HER2 heterodimers to lapatinib disruption in SKOV3 cells and increased sensitivity in H1650 cells, with reciprocal effects observed for HER1 homodimerization, may reflect lapatinib's potency to block relatively low, but not high, levels of HER1-HER2 or HER1 homodimers that are cell line- and HER level-dependent (Figure 2). On the other hand, erlotinib, a more selective HER1 tyrosine kinase inhibitor, stabilized EGF-dependent HER1-HER2 heterodimers in SKOV3 cells while having little effect on HER1 homodimerization (Figure 4c). These effects were similar in H1650 cells (Figure S3B in Additional file 1). In both cell lines, erlotinib suppressed EGF-dependent HER1 and HER2 phosphorylation, with HER1 phosphorylation more effectively inhibited relative to HER2, consistent with the reported HER1 selectivity of erlotinib. These results are consistent with several studies showing that HER1 tyrosine kinase inhibitors may stabilize inactive HER1-containing dimers, most notably gefitinib binding to the open or active HER1 kinase conformation [46-48]. Here, EGF-dependent HER1-HER2 heterodimers were stabilized by erlotinib but no effect on HER1-HER1 homodimers was observed (Figure 4c), which may represent a specific drug action of erlotinib.

**Figure 5:**
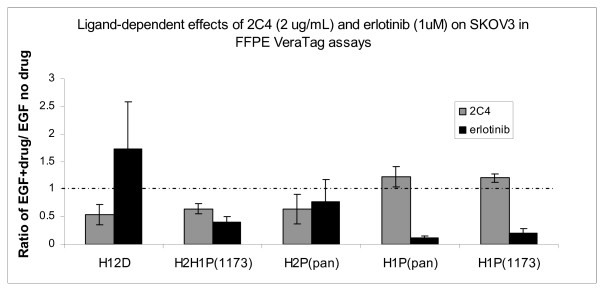
**EGF-dependent effects of 2C4 and erlotinib measured by VeraTag™ FFPE assays**. SKOV3 cells were treated as described for Figure 4, but cells were fixed and made into FFPE blocks instead of lysed. Each VeraTag™ FFPE assay, listed on the X-axis, was performed on six slides. Results are depicted as the ratio of the normalized RPA of EGF and drug-exposed cells to the normalized RPA of cells that were exposed to EGF alone. Gray bar: 2C4. Black bar: Erlotinib. Values above the dashed line at ratio = 1 represent stabilization due to drug exposure, and values below the dashed line represent inhibition due to drug exposure.

The dynamic changes in the levels of EGF-dependent HER1-HER2 heterodimers supported by 2C4 inhibition and erlotinib stabilization were utilized to establish the validity of these measurements in FFPE tumor cell lines. SKOV3 cells were exposed to either 2C4 or erlotinib and EGF as described above, fixed and made into FFPE blocks, and measurements were made by VeraTag™ FFPE assays. Consistent with results from the lysate assays, EGF-dependent HER1-HER2 heterodimerization was suppressed whether measured using the HER1-HER2 or the pHER1-HER2 assay. A decrease in HER2 phosphorylation was detected with the pHER2(pan) assay, while minimal change was observed in HER1 phosphorylation as detected by either the pHER1(pan) or pHER1(1173) assays (Figure 5). Upon erlotinib exposure, phosphorylation of HER1 was diminished as evidenced by the decrease in signal in pHER1(pan), pHER1(1173) and pHER1-HER2 assays. Inactive HER1-HER2 heterodimers were stabilized as observed in the lysate assays. A significant decrease in HER2 phosphorylation was not detected, in contrast with results from the lysate assay in which a twofold decrease was observed (Figure 4). This may be a reflection of a somewhat lower sensitivity of drug effects measured by the FFPE-based VeraTag™ assay, relative to the VeraTag™ lysate assay. Taken together, these results indicate that several dynamic changes in HER1-HER2 heterodimerization and activation promoted by HER targeted drugs and observed in cell lysates can also be detected in FFPE tumor cells by specifically designed VeraTag™ assays.

### Analysis of the HER1-HER2 axis in high HER2 breast tumors

To determine the prevalence of HER1 and HER1-HER2 dimeric and activated forms in HER2-positive breast cancer, 43 breast tumors that were pre-selected for high HER2 expression by IHC and subsequent scoring using the Allred system [49] (Asterand, Detroit, MI, USA) were analyzed by HER1-HER2 VeraTag™ FFPE tissue assays. Of these tumors, 39 were HER2 positive by the HERmark^® ^breast cancer assay [39], consistent with their independently determined Allred = 6 to 8 category scores. Total HER1 was measured in these tumors by VeraTag™ FFPE cell assay as described [38], with modifications (see Materials and methods). A total of 16 of the 39 FFPE tumors that were HER2 positive also displayed VeraTag™ HER1 signal that was greater than that of cell line MDA-MB-435, which expresses approximately 10,000 HER1 receptors/cell by flow cytometry (data not shown). A second format of the HER1 VeraTag™ assay, which utilized an antibody directed against the ECD rather than the ICD of HER1, was run on a subset of 30/43 tumors upon which macrodissection of non-tumor elements were performed (see Materials and methods). The ICD and ECD assays correlated significantly for both intact (R = 0.4885, *P *= 0.0113; Figure S4A in Additional file 1) and macrodissected samples (R = 0.5269, *P *= 0.0068; Figure S4B in Additional file 1). A comparison of intact tumor sections, containing representation of stroma, fat, tumor and normal epithelia, with the macrodissected sections in the ECD assay yielded nearly identical results (R = 0.8964, *P *< 0.0001; Figure S4C in Additional file 1). Taken together, these results suggest that the HER1 signal generated by the VeraTag™ assay was predominately derived from epithelial tumor cells in this sample set, although others have observed HER1 in normal epithelium [50]. There is general agreement between the tumor cell HER1 measured by IHC or HER1 VeraTag™ assays of macrodissected breast tumors (Figure S5A in Additional file 1). Analysis of HER1 IHC by a board certified pathologist further supported that the majority of the HER1 signal was derived from tumor cells rather than normal tissue (Figure S5C-J in Additional file 1).

Most HER1 and high HER2 expressing tumors expressed HER1 at low to moderate levels (IHC category 1+, 2+), whereas 2 of 43 samples that had high HER1 levels by VeraTag™ assay and HER1 IHC were not HER2-positive by HERmark^® ^(Figure S5B in Additional File 1). Although this suggests that HER1 is not highly co-expressed with HER2 when HER2 is overexpressed, it is consistent with the possibility of ligand-dependent and independent HER1-HER2 heterodimerization in HER2-positive breast cancer cells.

To evaluate the breast tumors with the HER1-HER2 heterodimer and activated assays, all samples were analyzed with a paired isotype control, and positives were classified as described in the Methods. Heterodimer signals in the tumors classified as positive were greater than or equal to that of cell line H1650 stimulated with 100 nM EGF. HER1-HER2 heterodimers were detected in 4 of the 16 tumors that were classified as positive for both HER1 and HER2, and pHER1-HER2 heterodimers were detected in 8 of these 16 tumors. The two heterodimer measurements were likely discrepant since the pHER1-HER2 assay appears to be more sensitive and may be able to detect lower levels of analyte.

**Figure 6:**
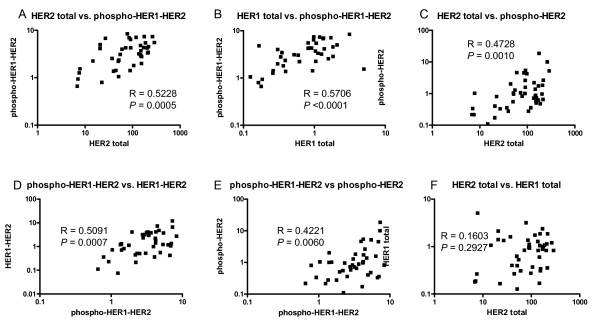
**Comparison of HER1/HER2 assay analytes in breast tumors pre-selected for HER2 positivity**. **A) **Phospho-HER2 correlations with total HER2. **B) **Phospho-HER1-HER2 correlates with total HER2. **C) **Phospho-HER1-HER2 correlates with total HER1. **D) **Phospho-HER1-HER2 correlates with HER1-HER2 backbone assay. **E) **Phospho-HER2 correlates with phospho-HER1-HER2. **F) **Correlation of total HER1 and total HER2 is not statistically significant. For all assay comparisons the total assay data in RPA*IB/TA without subtraction of isotype control background signal were used.

Different molecular species within the HER1/HER2 axis were compared to one another for possible relationships. Phospho-HER2 trended linearly with HER2 total measurements (Spearman R = 0.4728, *P *= 0.001) (Figure 6c). However, pHER2 measurements spanned a range of up to one log or greater for any given HER2 total level, supporting potential utility of pHER2 to further stratify HER2 positive tumors, if similar levels of phospho-preservation were achieved. Phospho-HER1-HER2 measurements correlated with HER1 (R = 0.5706, *P *< 0.0001) and HER2 (R = 0.5228; *P *= 0.005) (Figure 6a, b). HER1-HER2 and pHER1-HER2 measurements correlated significantly (R = 0.5091; *P *= 0.0007) (Figure 6d). The correlation between total HER1 and total HER2 was not significant (R = 0.1603, *P *= 0.2927) (Figure 6F). Similar results were obtained from data corrected for isotype control background (data not shown). Additionally, a subset of tumors having matched fresh frozen blocks were lysed and analyzed by Western blot for HER1 and HER2, or by co-IP/Western blots for pHER2 and pHER1-HER2 heterodimers (Figure S6 in Additional file 1). Results confirm the presence or absence of the different HER forms detected by VeraTag™ assays, with relative amounts generally consistent but some variation likely due to the semi-quantitative nature of Western blots, variable recovery yields from tissue lysis and co-immunoprecipitation, phosphoprotein and dimer stability versus FFPE preservation, and tumor heterogeneity within matched FFPE and fresh-frozen/lysed patient samples.

## Discussion

Using VeraTag™ technology, we have developed multiple quantitative and sensitive assays for measurements of activated receptor species in the HER1-HER2 signaling axis in both breast tumor cell lysates and FFPE cell and tumor formats. Using the VeraTag™ assay for cell lysates, control cell lines were identified which expressed different levels of HER1 and HER2, and generated a range of ligand-dependent and independent levels of phosphorylated HER proteins and HER1-HER2 heterodimers. These findings were confirmed by co-immunoprecipitation analysis. The cell lines were then utilized in the development and characterization of VeraTag™ assays that measured activated HER1/HER2 forms in FFPE tumor cells. Both the VeraTag™ lysate and FFPE cell assays demonstrated utility in the study of drug action and in the evaluation of activated HER1/HER2 forms in HER2-positive breast tumor tissue. Taken together, these studies indicate that VeraTag™ assays can be utilized in preclinical studies of drug mechanism of action involving both protein phosphorylation and protein-protein modes of signal activation, and has the potential for use in the *in situ *measurement of activated signal proteins in FFPE tumors toward biomarker identification in patient samples.

Several specific findings were made through the application of the VeraTag™ lysate assays. First, the levels of ligand-dependent and independent HER1-HER2 heterodimers appear to depend on the levels of HER1 and HER2. These results are consistent with recent studies indicating the existence of ligand independent HER1-HER2 and HER2-HER3 heterodimers [10-14,42,51] and suggest the possibility of elevated basal signaling in the presence of overexpressed HER receptors. Increased levels of ligand-independent HER1-HER2 heterodimers were also observed in high HER1 and HER2 expressing cell lines prepared by the cell lysate or FFPE cell formats. Although the levels of HER1-HER2 and pHER1-HER2 heterodimers display a dependency on HER1 and HER2 expression, a range of heterodimer signal is observed for a given level of HER1 or HER2. To the extent that levels of HER1-HER2 heterodimers may provide unique biological information beyond that of HER1 and HER2 expression together or alone will likely require clinical evaluation. Second, we made several novel observations regarding drug effects on the HER1/HER2 receptor axis. Pertuzumab, the humanized version of the monoclonal antibody 2C4 that binds to the dimerization arm of HER2 [43,52], has also been described as an inhibitor of HER2-HER3 heterodimerization, downstream signaling, and tumor cell growth [53]. More recent work has supported an additional role for 2C4 in inhibition of HER1-HER2 heterodimerization [44,45] although one group has shown 2C4 stabilization of this complex [54]. Results of the HER1-HER2 VeraTag™ lysate assay on SKOV3 and two other cell lines (H1650 and SKBR3), acutely exposed to 2C4 prior to EGF stimulation, were consistent with 2C4 acting as an inhibitor of HER1-HER2 heterodimerization. Intriguingly, an increase in HER1-HER1 homodimerization was observed in all three cell lines investigated, consistent with a shift in HER2 receptor availability for binding to HER1. EGF-dependent HER2 phosphorylation was decreased significantly while ligand-independent phosphorylation was unaffected, suggesting that transphosphorylation of HER2 by HER1 was inhibited by 2C4, while HER2 autophosphorylation or phosphorylation by other sources was not. These results are consistent with a pattern of ligand-activated HER2 heterodimerization inhibition by 2C4 [42-44,53].

Additional novel observations were made from VeraTag™ lysate analysis of drug effects of the 4-quinazoline tyrosine kinase inhibitors lapatinib and erlotinib. Lapatinib is a small molecule tyrosine kinase inhibitor (TKI) of both HER1 and HER2 [55], and erlotinib is a TKI that preferentially inhibits HER1 [56,57]. As expected, lapatinib exposure suppressed both HER1 and HER2 phosphorylation, supporting validity of VeraTag™ lysate assays to detect these measurements. Not only did erlotinib exposure suppress HER1 phosphorylation, but it also decreased EGF-dependent HER2 phosphorylation in a pattern similar to that of 2C4. This is consistent with loss of HER1-dependent HER2 transphosphorylation. In contrast to 2C4, however, erlotinib stabilized EGF-dependent HER1-HER2 heterodimers in all three cell lines investigated. These heterodimers were inactive since phosphorylation of both receptors was inhibited, with a preference for HER1 inhibition in high HER2 SKOV3 cells. These results support previous co-immunopreciptation and cross-linking studies suggesting that members of this quinazoline family of TKIs may stabilize inactive HER heterodimers while inhibiting phosphorylation and downstream signaling [46-48]. Mechanistic and structural studies indicate that erlotinib binds to the open, active conformation of HER1 kinase domain [58], while lapatinib binds to the closed, inactive conformation [55]. Here, erlotinib may have enabled trapping of HER1 in the open, active conformation that promotes HER dimerization as observed for gefitinib [48], leading to an increase in detectable but inactive HER1-HER2 heterodimers. Lapatinib, however, did not stabilize HER1-HER2 heterodimers. Intriguingly, lapatinib suppressed HER1-HER1 homodimerization in the HER2-overexpressing SKOV3 cell line. In contrast, in H1650 cells that have lower HER2 levels relative to SKOV3 cells and thus less HER1-HER2, HER1-HER2 heterodimers decreased but HER1-HER1 homodimer levels were unaffected. Since lapatinib inhibits both HER1 and HER2, we hypothesize that the discrepancy in dimerization inhibition pattern between SKOV3 and H1650 may be due to the proportion of HER1 and HER2 receptors. In cell lines that predominantly signal through HER1-HER2, the reduced levels of HER1-HER1 dimer and pHER1 may be more sensitive to inhibition by a dual kinase inhibitor at the concentrations tested here, while the opposite would be true in cell lines that signal predominantly through HER1-HER1 dimerization. Further experiments are needed to confirm this hypothesis.

VeraTag™ assays on FFPE cell line sections utilizing 2C4 and erlotinib were consistent with results from lysate assays and with expectations from published results, supporting utility and extending the use of these assays in understanding receptor activation in cell lysate and FFPE tumor tissues. To the best of our knowledge, this is the first example of HER1-HER2 heterodimer measurements made in FFPE tissue that have been characterized using drug inhibition, and suggests application to both cell-based and *in vivo *drug studies. Further, our studies have suggested interesting biology regarding HER1 and HER2 receptor switching. While findings are preliminary, they are suggestive of the ability of cell lines to redistribute signaling patterns between HER1-HER1 homodimers and HER1-HER2 heterodimers depending on relative HER levels, acute drug exposure and mechanism of drug action.

Last, we utilized the HER1/HER2 FFPE cell assays to determine the prevalence of HER1-HER2 dimers and phosphorylated forms in 43 breast tumor samples that were pre-selected for HER2-positivity. Of the 16 tumors that co-expressed HER1, 25% had detectable HER1-HER2 heterodimers whereas 50% had detectable pHER1-HER2. Phosphorylation of HER2 was detected in more than 50% of the HER2-positive tumors. Both autophosphorylation and transphosphorylation due to heterodimerization may contribute to overall HER2 phosphorylation, and both may be relevant in determining patient treatment. Historically, there has been a reluctance to measure phosphorylation in FFPE tissues due to the potential for phosphatase activity during tumor excision and time to fixation. However, recent technical advances to stabilize phosphorylation, such as decreased time to fixation and addition of phosphatase inhibitors to the fixative solution, along with increased evidence for the utility of phosphoprotein measurements, support development of technologies to specifically and quantitatively measure phosphoproteins in FFPE tissues. Phosphorylated HER2 has been shown to be a predictor of poor response and survival in breast cancer patients in multiple studies [59-61]. In a study of 816 archival FFPE breast cancer tissues, HER2-positive patients could be further stratified by pHER2 status, and those with pHER2 detected by IHC had lower survival rates than those that did not [62]. Tumors co-expressing pHER2 and pHER3 correlated with response to lapatinib in a Phase II clinical study of advanced inflammatory breast cancer patients [63]. Recently, a study utilizing Reverse Phase Protein Microarray identified a subset of breast tumors from the I-SPY clinical trial that were HER2 negative (unamplified) by Fluorescence *In Situ *Hybridization (FISH) and IHC, but showed disproportionately high pHER2 and downstream signaling [64]. Ongoing studies will determine clinical significance of this subset. Measurements of phosphorylated HER1, while not as prevalent in the majority of HER2-positive breast tumors, may also aid in prediction of response to targeted therapies in *de novo *or acquired Herceptin-resistant breast cancers as well as multiple additional cancer types, including squamous cell carcinoma of the head and neck, lung, gastric, and triple-negative breast cancers [65-71]. Studies utilizing pHER1 VeraTag™ FFPE assays in such cancers are currently underway in a subset of these cancer types.

In our study, great care was taken to preserve phospho-protein epitopes. All tumor tissues were acquired from a single commercial vendor that utilized a rigorous protocol for tumor collection, fixation, and storage to promote preservation of epitopes, consistent with the ASCO/CAP guidelines for preparation of breast tumor tissue for HER2 testing (see Methods). Additionally, an independent study performed under similar conditions in tumor cell xenografts showed complete preservation of pHER [72]. While it cannot be completely excluded, such rigor in collection and fixation protocols minimizes the potential for discrepancies in phospho-degradation. A recent study showed that although the total time for fixation influenced the retention of phosphoprotein epitopes detected by immunoassays, the phosphorylated signaling proteins pAkt and pERK were relatively stable for 30 to 80 minutes post excision when formalin fixation was facilitated by thin diameter core cuts (approximately 2 mm) compared to thicker resected tumor tissues (median length = 29 mm) [73]. Despite the decreased overall signal in the resected tissues, pAKT and pERK levels significantly correlated with levels measured in core cuts [73], suggesting that these phospho-measurements may have utility even under imperfect conditions. Thus, although complete retention of pHER may not have been achieved in the clinical studies just described above, or in the present study, sufficient preservation of pHER epitopes very likely occurred, since significant correlations were established with clinical outcome in the prior studies, and with other relevant VeraTag™ measurements in our study. Finally, while interpretation of the phosphoprotein data in this tumor set may be limited in terms of absolute value, our data clearly illustrate the ability of VeraTag™ technology to measure phospho-HER analytes in FFPE tissues, and can be tested for possible clinical utility if stringent control of cold ischemic time and phospho-epitope preservation is employed with patient samples.

In sum, the VeraTag™ lysate assays presented here may prove extremely useful when used in concordance with preclinical drug development, while FFPE assays may help fulfill the need for sensitive, quantitative methods for detection of activated HER1 and HER2 complexes in FFPE tumor tissues. With the advent of additional molecularly targeted therapies into the clinic, a thorough understanding of receptor activation and detection in patient samples has potential to enable the measurement of biomarkers that may be more predictive of drug response.

## Conclusions

We have developed novel, quantitative assays that measure HER1-HER2 dimerization and phosphorylation, HER1 phosphorylation, and HER2 phosphorylation in both lysate and FFPE tissue formats using VeraTag™ technology. The VeraTag™ assays were used to characterize the effects of 2C4, lapatinib, and erlotinib on cell lines, and receptor signaling switching between HER1-HER1 homodimers and HER1-HER2 heterodimers was observed under some conditions. Further, HER1-HER2 heterodimers were detected in a subset of HER2-positive breast tumors that co-expressed HER1. These VeraTag™ assays may have utility both in pre-clinical drug development and for predicting clinical response to targeted agents.

## Abbreviations

CE: capillary electrophoresis; Co-IP: co-immunoprecipitation; DMSO: dimethyl sulfoxide; ECD: extracelluar domain; EGF: epidermal growth factor; EGFR: epidermal growth factor receptor; FACS: fluorescent activated cell sorting; FISH: fluorescence in situ hybridization; FFPE: formalin-fixed: paraffin-embedded; IB: illumination buffer; IHC: immunohistochemistry; ITC: isotype control; PBS: phosphate-buffered saline; PBS-T: phosphate-buffered saline-Tween 20; PLA: proximity ligation assay; RPA: relative peak area; SA-MB: streptavidin-methylene blue; TK: Tyrosine kinase; TKI: Tyrosine kinase inhibitor.

## Competing interests

All authors are or were previously employed by Monogram Biosciences, Inc., which offers HERmark^® ^assays commercially for patient testing and uses VeraTag™ assays in collaborations with multiple private and academic institutions.

## Authors' contributions

LDE designed and oversaw all experiments, carried out original co-immunoprecipitiations and some VeraTag™ FFPE assays, and drafted the manuscript. KS carried out the majority of the VeraTag™ FFPE and lysate assays depicted here. TDP designed and made the transfected 293 clones, carried out the co-immunoprecipitation experiments depicted here, performed the drug experiments on SKOV3 cells, and carried out some VeraTag™ FFPE assays. GP provided intellectual input and helped edit the manuscript. LG provided intellectual input, helped edit the manuscript, and selected tumor sets. JW provided intellectual input, selected tumor sets, oversaw tumor immunohistochemistry, and helped edit the manuscript.

## Supplementary Material

Additional file 1 **Supplementary Figures S1 to S6 Figure S1. Ligand-dependent and ligand-independent HER1-HER2 heterodimerization depends on receptor levels. **A total of 293 cells were transfected with HER1 to generate 293H1_clone11. Clone11 was then co-transfected with HER2 to generate clones 12, 15, 16, and 19. Gray bars represent signal from mock-stimulated cells, and black bars represent signal from cells stimulated with 100 nM EGF. A) HER1 total lysate assay. All clones had the same amount of HER1. B) HER2 total lysate assay. Clones displayed different amounts of total HER2. C) HER1-HER2 heterodimer lysate assay. Ligand-dependent and ligand-independent HER1-HER2 heterodimer formation increased with increasing HER2 receptor number in clones with the same amount of HER1. **Figure S2. Specificity of clone 53A5 for phospho-HER1 in FFPE assay. **AU565 FFPE slides from cells were mock-stimulated or stimulated with 100 nM EGF for 10 minutes. The pHER1-HER2 FFPE assay was performed, but peptide was incubated with antibody in molar ratios 1:0, 1:1, 1:10, or 1:100 of antibody to peptide. Gray bars represent signal from mock-stimulated cells, and black bars represent signal from EGF-stimulated cells. Phospho-HER1-HER2 signal is competed to basal levels using the antigenic peptide H1pY1173 but not with the nonphosphorylated H1Y1173 nor the homologous HER2 peptides H2pY1248 nor H2Y1248. **Figure S3. Effects of lapatinib and erlotinib measured by VeraTag™ lysate assays in H1650. **H1650 cells were serum-starved overnight then treated with lapatinib or erlotinib for two hours at the indicated concentrations, in units of μM. A final concentration of 16 nM EGF was added or mock-added to the drug-containing media for 10 minutes prior to harvesting cells for lysate or FFPE. VeraTag™ lysate assays were used to examine A) effects of erlotinib and B) effects of lapatinib on HER1-HER2 heterodimerization (top row), phosphorylation of HER2 (second row), phosphorylation of HER1 (third row), and HER1-HER1 homodimerization (bottom row). **Figure S4. Comparison of two different formats of a HER1 total protein VeraTag™ assay performed in HER2+ breast FFPE tumors. A) **Comparison of a HER1 ICD-directed total protein assay with a HER1 ECD-directed total protein assay, both applied to intact FFPE tumor sections. **B) **Comparison of a HER1 ICD-directed total protein assay performed in intact FFPE tumor sections with a HER1 ECD-directed total protein assay having non-tumor elements macrodissected from the FFPE tumor sections. **C) **Comparison of HER1 values measured in intact versus macrodissected FFPE tumor sections assayed by the HER1 ECD-directed total protein assay. **Figure S5. Comparison of HER1 ECD-directed total protein assay with HER1 IHC H-score. A) **A subset of 30 HER2+ breast tumors was assayed by a HER1 ECD-directed total protein assay with non-tumor elements macrodissected away, and the HER1 levels were compared with the tumor cell HER1 IHC H-scores measured on adjacent sections. The IHC H-score spans 0 to 300. Tumors with an H-score of 0 were assigned a score of 0.1 to make plotting on a log scale possible. **B) **The percentage of the 30 tumors at each HER1 IHC test category. **C-F) **Micrographs of the HER1 IHC staining representing four separate invasive ductal carcinoma breast tumors that are representative of other tumors having similar staining intensity from IHC = 3+ to 0, respectively (4× magnification). **G-J**) Same tumor micrographs as in C-F but at 20× magnification. **Figure S6. Western blot and co-IP/Western blots of lysed tumor tissue homogenates. **Fresh frozen tumors matched with FFPE blocks of HER2+ breast tumors were lysed and homogenized with protease and phosphatase inhibitors, and analyzed by SDS-PAGE and Western blots. Sections of the matched FFPE blocks were assayed by the comparable VeraTag™ assay. **A) **HER2 Western blot comparison of tumor lysate with HER2 total protein VeraTag™ assay. **B) **HER1 Western blot of tumor lysate comparison with HER1 ECD-directed total protein VeraTag™ assay. **C) **Tumor lysate immunoprecipitated with a HER2 antibody and Western blot with a pan-phosphotyrosine antibody comparison with pHER2 VeraTag™ assay. **D) **Tumor lysate immunoprecipitated with pHER1(1173) antibody and Western blot with a HER2 antibody comparison with pHER1-HER2 VeraTag™ assay. Click here for file
