# Supplementary material for: Quantitative assays for the measurement of HER1-HER2 heterodimerization and phosphorylation in cell lines and breast tumors: applications for diagnostics and targeted drug mechanism of action
Source: Breast Cancer Res. 2011 Apr 15;13(2):R44. doi: 10.1186/bcr2866 (PMC3219207; doi:10.1186/bcr2866)
Supplement: Additional file 1 — Supplementary Figures S1 to S6 Figure S1. Ligand-dependent and ligand-independent HER1-HER2 heterodimerization depends on receptor levels. A total of 293 cells were transfected with HER1 to generate 293H1_clone11. Clone11 was then co-transfected with HER2 to generate clones 12, 15, 16, and 19. Gray bars represent signal from mock-stimulated cells, and black bars represent signal from cells stimulated with 100 nM EGF. A) HER1 total lysate assay. All clones had the same amount of HER1. B) HER2 total lysate assay. Clones displayed different amounts of total HER2. C) HER1-HER2 heterodimer lysate assay. Ligand-dependent and ligand-independent HER1-HER2 heterodimer formation increased with increasing HER2 receptor number in clones with the same amount of HER1. Figure S2. Specificity of clone 53A5 for phospho-HER1 in FFPE assay. AU565 FFPE slides from cells were mock-stimulated or stimulated with 100 nM EGF for 10 minutes. The pHER1-HER2 FFPE assay was performed, but peptide was incubated with antibody in molar ratios 1:0, 1:1, 1:10, or 1:100 of antibody to peptide. Gray bars represent signal from mock-stimulated cells, and black bars represent signal from EGF-stimulated cells. Phospho-HER1-HER2 signal is competed to basal levels using the antigenic peptide H1pY1173 but not with the nonphosphorylated H1Y1173 nor the homologous HER2 peptides H2pY1248 nor H2Y1248. Figure S3. Effects of lapatinib and erlotinib measured by VeraTag™ lysate assays in H1650. H1650 cells were serum-starved overnight then treated with lapatinib or erlotinib for two hours at the indicated concentrations, in units of μM. A final concentration of 16 nM EGF was added or mock-added to the drug-containing media for 10 minutes prior to harvesting cells for lysate or FFPE. VeraTag™ lysate assays were used to examine A) effects of erlotinib and B) effects of lapatinib on HER1-HER2 heterodimerization (top row), phosphorylation of HER2 (second row), phosphorylation of HER1 (third row), and HER1-HER1 homodimer [file bcr2866-S1.PDF]

Figure S1

A

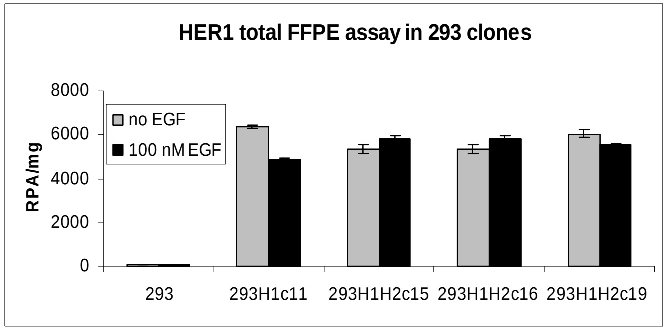

B

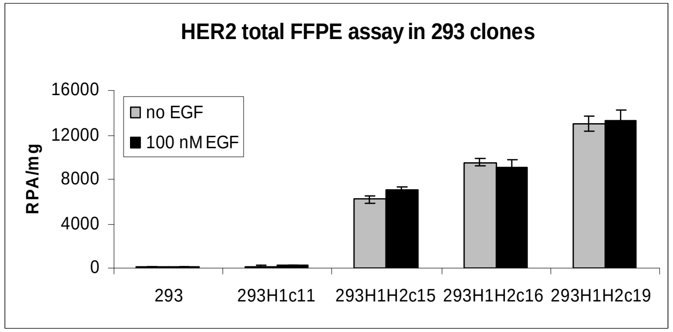

C

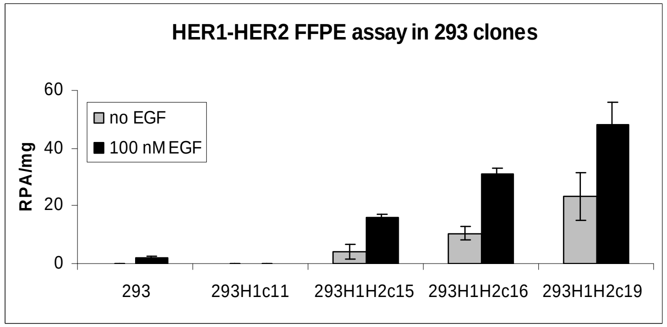

Figure S2

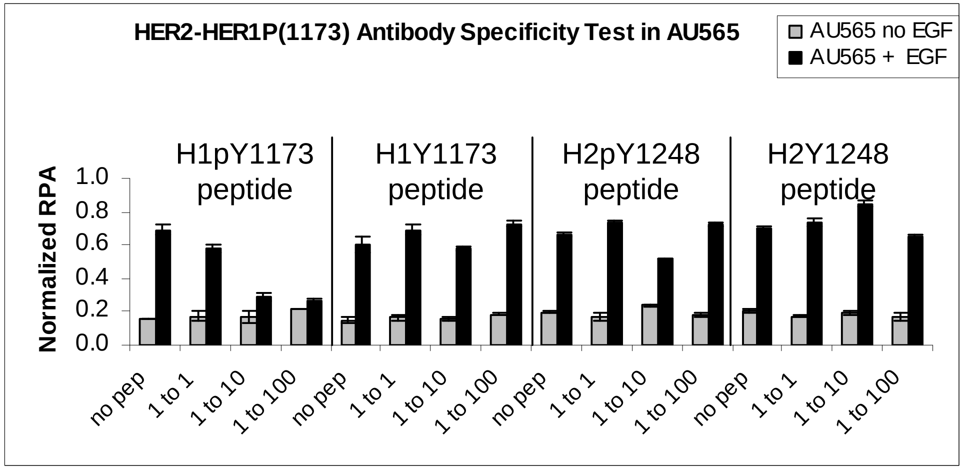
HER2-HER1P(1173) Antibody Specificity Test in AU565
AU565 no EGF
AU565 + EGF
Normalized RPA
1.0
0.8
0.6
0.4
0.2
0.0
H1pY1173 peptide
H1Y1173 peptide
H2pY1248 peptide
H2Y1248 peptide
no pep
1 to 1
1 to 10
1 to 100

Figure S3

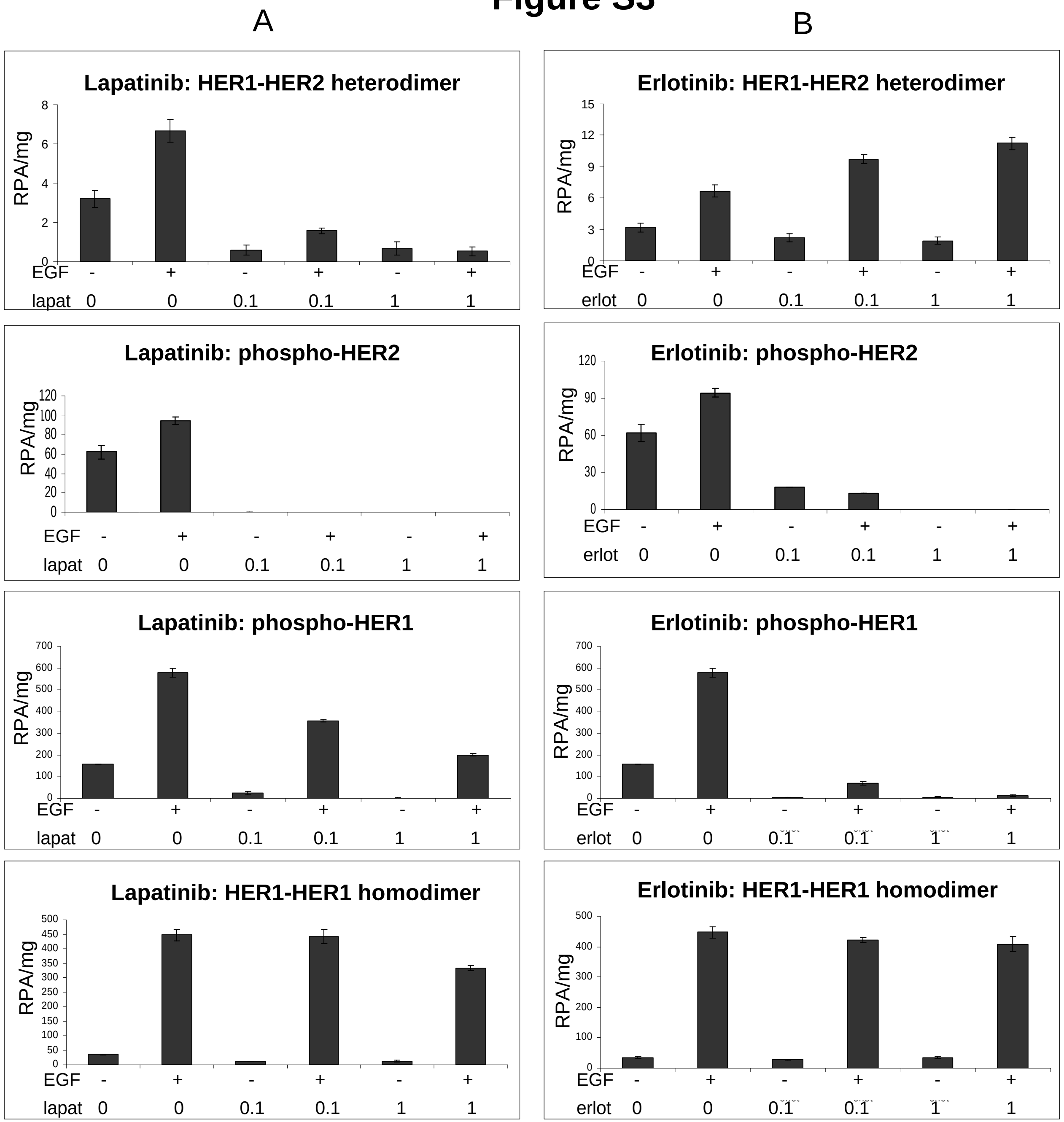

A
B
Lapatinib: HER1-HER2 heterodimer
RPA/mg
0
2
4
6
8
EGF - + - + - +
lapat 0 0 0.1 0.1 1 1
Erlotinib: HER1-HER2 heterodimer
RPA/mg
0
3
6
9
12
15
EGF - + - + - +
erlot 0 0 0.1 0.1 1 1
Lapatinib: phospho-HER2
RPA/mg
0
20
40
60
80
100
120
EGF - + - + - +
lapat 0 0 0.1 0.1 1 1
Erlotinib: phospho-HER2
RPA/mg
0
30
60
90
120
EGF - + - + - +
erlot 0 0 0.1 0.1 1 1
Lapatinib: phospho-HER1
RPA/mg
0
100
200
300
400
500
600
700
EGF - + - + - +
lapat 0 0 0.1 0.1 1 1
Erlotinib: phospho-HER1
RPA/mg
0
100
200
300
400
500
600
700
EGF - + - + - +
erlot 0 0 0.1 0.1 1 1
Lapatinib: HER1-HER1 homodimer
RPA/mg
0
50
100
150
200
250
300
350
400
450
500
EGF - + - + - +
lapat 0 0 0.1 0.1 1 1
Erlotinib: HER1-HER1 homodimer
RPA/mg
0
100
200
300
400
500
EGF - + - + - +
erlot 0 0 0.1 0.1 1 1

Figure S4

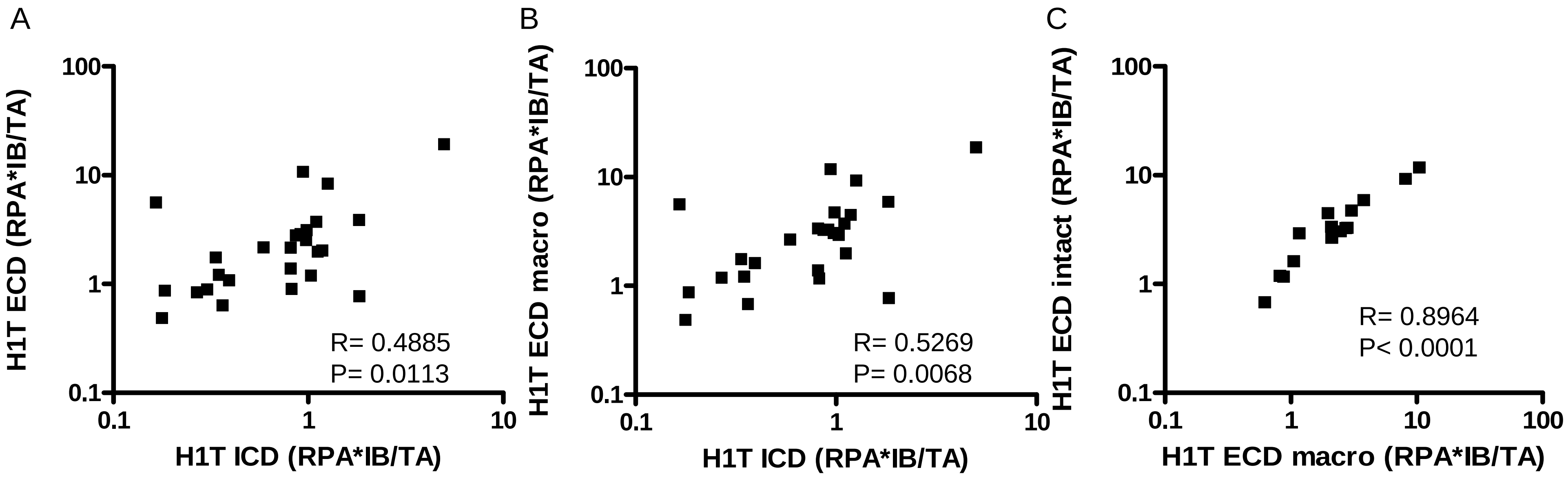
A
H1T ECD (RPA*IB/TA)
100
10
1
0.1
0.1
1
10
H1T ICD (RPA*IB/TA)
R= 0.4885
P= 0.0113
B
H1T ECD macro (RPA*IB/TA)
100
10
1
0.1
0.1
1
10
H1T ICD (RPA*IB/TA)
R= 0.5269
P= 0.0068
C
H1T ECD intact (RPA*IB/TA)
100
10
1
0.1
0.1
1
10
100
H1T ECD macro (RPA*IB/TA)
R= 0.8964
P< 0.0001

Figure S5

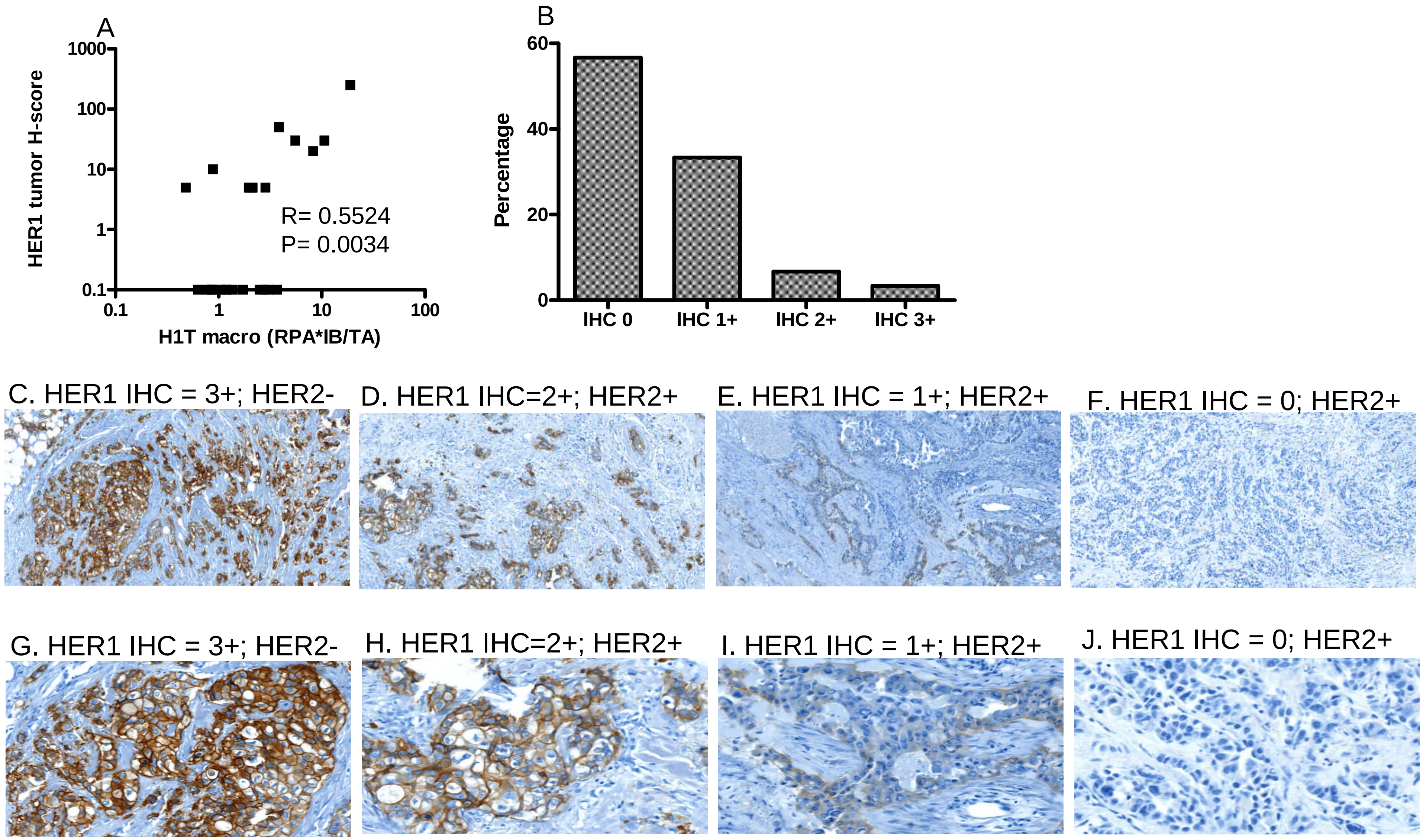
A
HER1 tumor H-score
1000
100
10
1
0.1
0.1
1
10
100
H1T macro (RPA*IB/TA)
R= 0.5524
P= 0.0034
B
Percentage
60
40
20
0
IHC 0
IHC 1+
IHC 2+
IHC 3+
C. HER1 IHC = 3+; HER2-
D. HER1 IHC=2+; HER2+
E. HER1 IHC = 1+; HER2+
F. HER1 IHC = 0; HER2+
G. HER1 IHC = 3+; HER2-
H. HER1 IHC=2+; HER2+
I. HER1 IHC = 1+; HER2+
J. HER1 IHC = 0; HER2+

Figure S6

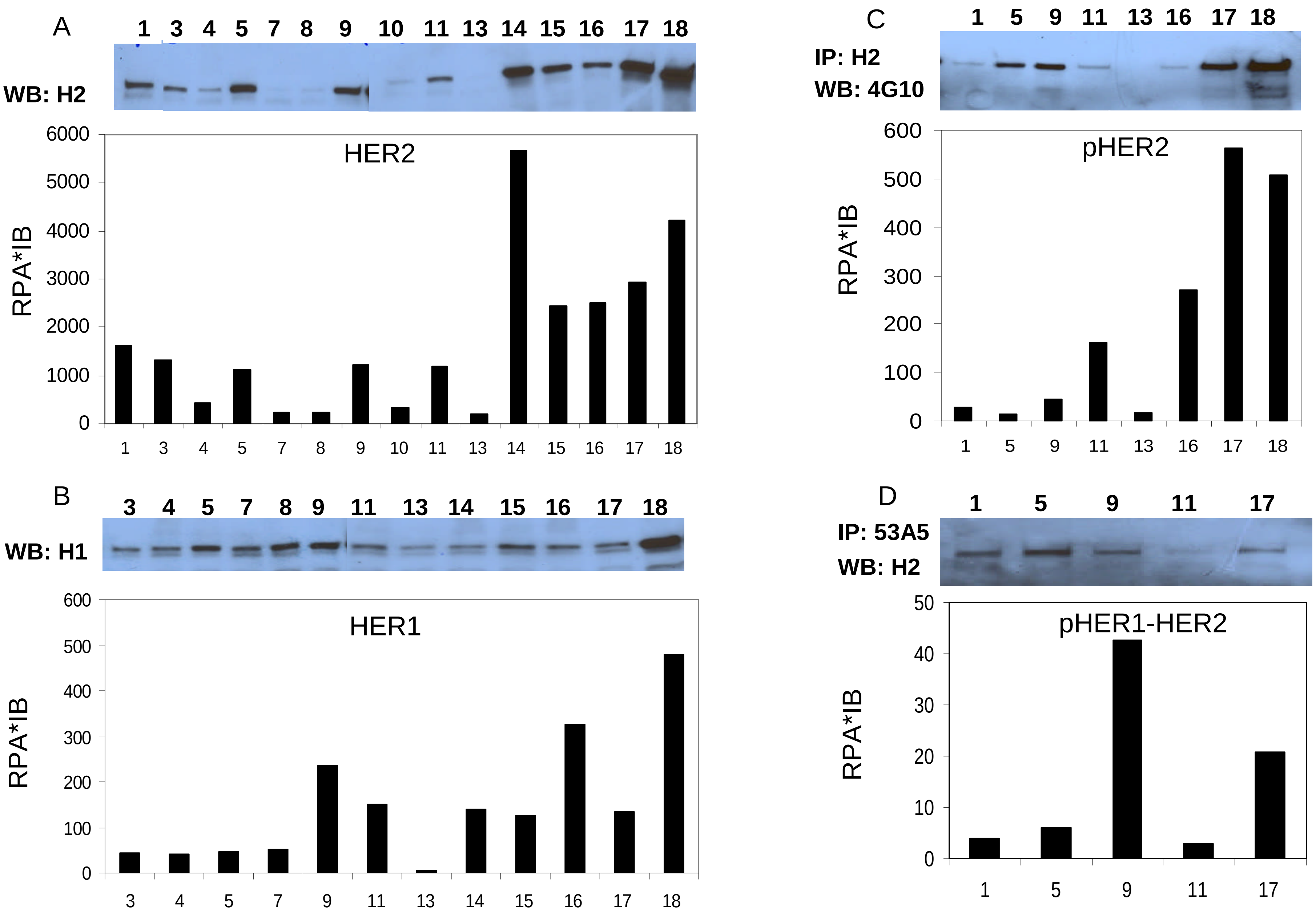

A
1 3 4 5 7 8 9 10 11 13 14 15 16 17 18
WB: H2
HER2
RPA*IB
0 1000 2000 3000 4000 5000 6000
1 3 4 5 7 8 9 10 11 13 14 15 16 17 18
B
3 4 5 7 8 9 11 13 14 15 16 17 18
WB: H1
HER1
RPA*IB
0 100 200 300 400 500 600
3 4 5 7 9 11 13 14 15 16 17 18
C
1 5 9 11 13 16 17 18
IP: H2
WB: 4G10
pHER2
RPA*IB
0 100 200 300 400 500 600
1 5 9 11 13 16 17 18
D
1 5 9 11 17
IP: 53A5
WB: H2
pHER1-HER2
RPA*IB
0 10 20 30 40 50
1 5 9 11 17
